# Cell therapies for pancreatic beta-cell replenishment

**DOI:** 10.1186/s13052-016-0273-4

**Published:** 2016-07-11

**Authors:** Bernard Okere, Laura Lucaccioni, Massimo Dominici, Lorenzo Iughetti

**Affiliations:** Division of Pediatric Oncology, Hematology and Marrow Transplantation, Department of Medical and Surgical Sciences for Children & Adults, University of Modena and Reggio Emilia, Modena Policlinic, Modena, 41100 Italy; Child Health, School of Medicine, Dentistry & Nursing, University of Glasgow, Glasgow, UK; Division of Oncology, Department of Medical and Surgical Sciences for Children & Adults, University of Modena and Reggio Emilia, Modena Policlinic, Modena, 41100 Italy

**Keywords:** Diabetes, Regenerative medicine, Cell therapy, Insulin-producing cells, Stem cells

## Abstract

The current treatment approach for type 1 diabetes is based on daily insulin injections, combined with blood glucose monitoring. However, administration of exogenous insulin fails to mimic the physiological activity of the islet, therefore diabetes often progresses with the development of serious complications such as kidney failure, retinopathy and vascular disease. Whole pancreas transplantation is associated with risks of major invasive surgery along with side effects of immunosuppressive therapy to avoid organ rejection. Replacement of pancreatic beta-cells would represent an ideal treatment that could overcome the above mentioned therapeutic hurdles. In this context, transplantation of islets of Langerhans is considered a less invasive procedure although long-term outcomes showed that only 10 % of the patients remained insulin independent five years after the transplant. Moreover, due to shortage of organs and the inability of islet to be expanded *ex vivo*, this therapy can be offered to a very limited number of patients. Over the past decade, cellular therapies have emerged as the new frontier of treatment of several diseases. Furthermore the advent of stem cells as renewable source of cell-substitutes to replenish the beta cell population, has blurred the hype on islet transplantation. Breakthrough cellular approaches aim to generate stem-cell-derived insulin producing cells, which could make diabetes cellular therapy available to millions. However, to date, stem cell therapy for diabetes is still in its early experimental stages. This review describes the most reliable sources of stem cells that have been developed to produce insulin and their most relevant experimental applications for the cure of diabetes.

## Background

The therapeutic approach for type 1 diabetes (T1D) is based on daily insulin injections, combined with blood glucose monitoring, healthy lifestyle and diet. Administration of exogenous insulin fails to mimic the physiological activity of the islet and disease progression may lead to the development of serious complications such as kidney failure, retinopathies and vascular diseases.

Replacement of pancreatic beta-cells represents an ideal treatment that could overcome the above mentioned therapeutic hurdles. Whole pancreas transplantation was performed for the first time in 1966 by Kelly and co-workers [1]. It is currently performed in association with kidney transplantation in adult patients with both renal failure and T1D. Although the positive 1st year survival rates (77 %), the pancreas transplant is associated with the risks of any major invasive surgery along with the side effects of immunosuppressive therapy to avoid organ rejection.

In this context transplantation of islets of Langerhans is a less invasive procedure: after the enzymatic and mechanic isolation from the deceased donor’s organ, islets cells are purified and injected into the recipient’s liver via the portal vein, where they rapidly engraft and re-vascularize. Progresses in islet isolation protocols and immunosuppression therapy allowed restoration of normoglycemia in patients who received islets from multiple donors. The “Edmonton Protocol” is a milestone in this field and it is internationally considered the best procedure to explore feasibility and reproducibility of islet transplantation. Patients affected by T1D underwent islet transplantation in conjunction with a glucocorticoid-free immunosuppressive regimen consisting of sirolimus, tacrolimus,and daclizumab with a good outcome at 12 months of follow-up [2]. Although this innovative therapeutic approach was initially shown to be successful, long-term outcomes depicted a different scenario as about 10 % of the patients remained insulin independent five years after the transplant, although this is variable from a study to another. An international, multicentre trial conducted in 9 international sites and involving 36 patients with T1D, showed that 44 % achieved an insulin independence with adequate glycaemic control 1 year after the final transplantation (5 of them for 2 years), 28 % had partial function, and 28 % had complete graft loss 1 year after the final transplantation [3]. Brennan et al. reported the long-term (12 years) follow-up of the efficacy and safety of the first 7 islet transplantation in type 1 diabetic subjects. All seven subjects demonstrated continued islet function longer than a decade from the time of first islet transplantation. One subject remained insulin independent without the need for diabetic medications or supplemental transplants. At trial completion, five subjects were receiving insulin and two remained insulin independent, although one was treated with liraglutide. No patients experienced severe hypoglycaemia, opportunistic infection, or lymphoma [4]. Recently, long term follow up of two patients treated with Islet transplant according to the Edmond protocol in 2001 were reported as insulin-independent for two years [5], while the all the 10 patients described by Qui et al. in 2014 achieved an insulin independence after 1-3 transplants and at 5 years of follow-up, and 6 of them were free of exogenous insulin [6].

Although the Islet transplantation according to the Edmond Protocol showed good results in terms of safety, due to shortage of organs and inability of islet to be expanded ex vivo, this therapy can be offered to a very limited number of patients only [7]. However the Islet Transplant continues to be matter of interesting new studies, seen the recent results published by Berman et al. aiming to develope a clinically applicable protocol for extrahepatic transplantation of pancreatic islets. The potency of islets implanted onto the omentum, using an in situ–generated adherent, resorbable plasma-thrombin biologic scaffold, was evaluated in diabetic animal models. An improved metabolic function and preservation of islet cytoarchitecture was found and long-term nonfasting normoglycemia and adequate glucose clearance were achieved in both intrahepatic and intraomental sites in rats [8]. New promising results are also available on the protection that antiaging glycopeptide may offer to the Human Islets against Tacrolimus-Related injury facilitationg the engraftment in animal models [9].

Moreover, recent data were presented on the outcomes of Pancreas Transplantation procedure after Islet Transplantation failure (PAI) and vice-versa (IAP). Although only a very limited number of patients received these procedure, metabolic outcomes seems promitting, specially in PAI [10]. The potential for long-term banking of human islets for research, was also recently described. which could enable the use of tissue from a large number of donors with future technologies [11]. As described, despite Islet and Pancreas transplant is an important field in T1D treatment, over the past decade cellular therapies have emerged as the new frontier for the treatment of various lethal diseases and the advent of stem cells as a renewable source of cell-substitutes, to replenish the beta cell population, has blurred the hype on islet transplantation. Breakthrough cellular approaches aim to generate stem cell derived insulin producing cells, which could make diabetes cellular therapy available to millions. However, to date, stem cell therapy for diabetes is still in its early, yet promising, experimental stages.

## Stem cells: proprieties and classification

Stem cells are characterized by two key features: (i) as unspecialized cells they can divide and renew themselves for long periods without differentiating in other cell types, and (ii) they present the potential to develop into many specialized cell types, under certain physiologic or experimental conditions. Such process can be induced by different signals, either internal, as genes expression, or external, as physical contact, chemical secretions or micro-environmental molecules. The induction leads to cell specialization through the restriction and expression of key genes.

Stem cells can be classified in different levels by their plasticity, which is the ability to differentiate into the totality or just a part of cell lineages. The zygote, resulting by gametes fusion, represents a totipotent cell type, since it gives rise to both embryo and placental trophoblast. After four days of mitotic divisions, the zygote grows into a group of cells referred to as blastocyst. Embryonic stem cells (ESCs) deriving from the inner cell mass of the blastocyst are pluripotent, because of their ability to develop into the three germ layers but not into extraembryonic tissues. ESCs were first derived from mouse embryos [12] while in 1998 ESCs were isolated and cultured from human blastocysts. The first transplants of human ESCs in immune-deficient mice revealed an additional peculiarity of pluripotent stem cells: the capacity to generate teratomas composed of tissues from all lineages. In contrast, adult stem cells, identified in various organs and tissues, can be multipotent, showing the potential to differentiate into multiple cell lineages, or unipotent, when they retain a restricted ability to differentiate [13]. Because of their peculiarities, stem cells are considered the most promising candidates for future therapeutic approaches of T1D and other degenerative diseases. The aim of regenerative medicine for diabetes is to develop a renewable and safe source of stem cells to replenish the damaged cells, providing the patients with a long term source of insulin producing cells.

## Stem cell approaches to restore insulin production

### Adult stem cells: endodermic cells from pancreas and liver

Adult stem cells are undifferentiated cells showing the potential to self-renew and differentiate into one or few specific cell lineages of the tissue in which they are found or “neighbour” tissues, in order to maintain and repair the organ of origin. In situation of serious damages, various adult stem cell types can be recruited at distance to repair injured areas. For these reasons, adult stem cells are considered a suitable source for a wide range of cell therapy applications, due to their ability to differentiate *in vivo*, or to expand and differentiate *in vitro* their progeny [14].

#### Reprogramming pancreatic adult stem cells

Pancreatic ducts, exocrine pancreas and islet of Langerhans are proposed as sources of pancreatic stem/progenitor cells. Although their nature and even their existence were initially subject of controversy in the field of beta-cell replacement for diabetes [15], pancreatic resident adult stem cells have been successfully differentiated into islet-like cells. Studies on human pancreatic duct cells have shown their ability to both proliferate *in vitro* and differentiate into insulin producing cells [16, 17]. Other studies on pancreatic resident adult stem cells describe how, after partial pancreatectomy in diabetic mice, ductal progenitors are capable of generating mature ductal epithelial cells. The proliferation and differentiation of pancreatic progenitor cells, located in the pancreatic ductal epithelium, might be involved in this process. This hypothesis is suggested by the observation that, after pancreatectomy, proliferation starts from the main ducts followed by small ducts, until newly formed islets appear at the periphery of ductules at the final stage of regeneration [18–20]. Further experiments on human pancreatic duct cells have confirmed the outcomes from animal models. In particular external stimuli, such as extracellular matrix, enhance the expansion of ductal tissue and the differentiation to islet-like structures along with the production of insulin [21–23]. Moreover ductal cells after differentiation are able to re-express the key transcription factor IPF-1/PDX1 (insulin promoter facto-1/pancreas and duodenal homebox-1) [24], which plays a key role in pancreas development.

Attempts at producing islet cells from acinar cells were performed by Zhou et al. on rodent models. Their studies have provided direct evidence that insulin secreting cells can be generated *in vitro* from re-programming of differentiated cells with exocrine functions such as amylase/elastase-expressing pancreatic acinar cells and non-endocrine epithelial cells, this latter resulting from the remaining fraction after islet isolation [25].

Human pancreatic islets also contain an unrecognized distinct population of cells that expresses the neural stem cell-specific marker nestin. These promising nestin-positive cells isolated from adult pancreas of rodents and human, retain some proliferative capacity and can differentiate ex vivo into pancreatic exocrine and endocrine phenotypes [26].

#### Trans-differentiation of endodermic cells from liver

Liver and small intestine cells share the same lineage origin of pancreatic cells. Therefore, both have been tested as replacement insulin producing cells for diabetes cellular therapy [27–33].

To date, most of the approaches are focused on liver as a promising abundant source for the generation of insulin producing cells. Liver and pancreas cells, in vertebrates, derive from the same indistinct pool of progenitor stem cells resident in the extra-hepatic biliary tree [34] and differentiate into hepatic and pancreatic definitive tissues after chemical signals secreted by the developing heart. [35–39] The discovery of mechanisms for glucose sensing and signal transduction [40, 41] in liver triggered the first cell trans-differentiation assays to obtain insulin producing cells from hepatocytes. Several groups successfully transdifferentiated hepatic cells into insulin producing cells in rat models using adenovirus-mediated gene transfer approaches to insert PDX1 alone or in combination with NeuroD and MafA genes [42–45]. Despite the positive outcomes of these approaches, the clinical translation of methods to the human is not applicable because of the safety issues raised by the use of adenovirus. Furthermore, *in vitro* trans-differentiation led to hybrid hepatocyte-beta cell phenotype incapable of adjusting insulin levels according to variable glucose concentrations. Therefore, in the years to come further evaluations are required to make liver cells a concrete and viable source for beta cell replacement [46].

### Adult stem cells: haemopoietic, mesenchymal cells and pancreatic resident mesenchymal cells

#### The role of haemopoietic stem cells

Haemopoietic stem cells are situated in stem cell niches like bone marrow or umbilical cord blood and differ from mesenchymal cells, which are distributed in the entire body and can generate fibroblast, adipocyte, chondrocyte along with other connective cells [47]. Haemopoietic stem cells are primarily used to treat immune-related disorders because of their ability to encourage and stimulate vascular regeneration rather than an effective differentiation into insulin producing cells. Voltarelli et al. have described the attempt of resetting the immunological conditions of diabetes by autologous transplantation of hematopoietic bone marrow-derived stem cells, after high doses of immunosuppressive drugs. Results have shown that their attempt increased beta-cell function and prolonged insulin independence in the majority of the patients, increasing C-peptide levels and reducing hemoglobin A1c. However, the exact mechanism of action of autologous transplantation of bone marrow-derived stem cells in autoimmune disorders is not yet fully understood [48]. Other groups have injected hematopoietic bone marrow stem cells directly into pancreatic tissue of patients with type 2 diabetes, in combination with hyperbaric oxygen treatment. Such procedures have obtained promising outcomes, reducing insulin requirements and improving glycaemic control [49].

#### Mesenchymal stem cells

Ubiquitous mesenchymal stem cells (MSCs) can be isolated from various tissues like umbilical cord, bone marrow, adipose tissue and placental tissues. However, to be considered as MSCs, they must meet specific requirements such as adherence to plastic, differentiation abilities towards bone, cartilage and fat lineage, presence/absence of peculiar surface markers. Some MSCs also express stemness markers of pluripotency like OCT4 and Nanog [50]. Moreover, MSCs are characterized by high expansion potential and no tumorigenic risks [47–51]. MSC have shown, both *in vitro* and *in vivo*, a strong immunomodulatory activity which may help avoiding post-transplant rejection. Immunomodulation is mediated by different mechanisms: secretions of soluble factors (e.g. prostaglandin E2 and IL-10), pathways activated by direct contact with T-cell or interaction with dendritic cells [52]. Successful studies on human MSC differentiated into insulin producing cells were reported by Wu XH et al. [53] who demonstrated the capacity of bone marrow mesenchymal stem cells (BM-MSCs) to transdifferentiate *in vitro* into islet-like cells and transplanted the cells into streptozotocin-induced diabetic rat. Few years later in a similar attempt, Phadnis SM et al. failed to accomplish a sufficient *in vitro* differentiation of BM-MSC, while achieved the amelioration of glycemia after injection of immature cells in diabetic mice, showing that pre-induced BM- MSCs (*in vitro*) completed their maturation into endocrine pancreatic lineage and insulin producing cells *in vivo* [54]. Other studies describe protocols for generation of glucose-responsive insulin-producing islet-like clusters from accessible and abundant tissues like human adipose tissue (AD- MSCs) [55, 56]; from Wharton’s jelly [57],(WJ- MSCs), amniotic fluid (AF-MSCs) [58] and umbilical cord blood (UCB-MSC) [59]. Seeberg et al. have proposed to use mesenchymal stem cells from pancreas to produce beta-like cells, hypothesizing that the pancreatic origin would ease the induction process. Data have shown that MSCs isolated from adult human exocrine pancreatic tissue express the same cell surface antigens of MSCs derived from bone marrow, adipose tissue and umbilical cord blood, and the same ability to differentiate into mesodermic and endodermic cell lineages. Using a step-wise differentiation protocol they generated cells retaining a gene expression pattern (PDX1, Pax4 and ngn3) typical of beta cells [60–62]. Recently, it was also showed by Cal et al. the safety and the moderate improvement of metabolic effects on insulin secretion of umbilical cord (UC) MSCs plus autologous bone marrow mononuclear cell (aBM-MNC) stem cell transplantation (SCT) without immunotherapy in established type 1 diabetes (T1D) [63].

However, it is important to remark the limitations observed using MSCs to derive insulin producing cells. First, C-peptide levels of differentiated cells were low and unlikely to sustain normoglycemia in diabetic mice, indicating the necessity to improve, qualitatively and quantitatively, the insulin producing cell mass. On this regard, the different germ layer derivation of MSCs hinders the definition of differentiation protocols for insulin producing cells [12, 47]. Furthermore, MSCs clones display variable proliferation rates, which hamper the setting of standard protocols to obtain beta-like cells [51–62].

## Cellular therapy for type 1 diabetes with embryonic stem cells

Among the variety of stem cell types, embryonic stem cells (ESCs) retain the highest plasticity. ECSs can differentiate to all body cell lineages and are uniquely capable of indefinite self-renewal.

Applications of ES cells to human therapy began with their characterization by Thomson et al. (1998) [13]. In 2001 Lumelsky et al. described what seemed to be a simple method to obtain insulin-producing beta cells from human ESCs [64], although few years later it was demonstrated that such method was generating neuroectodermal cells instead of insulin-producing cells, with an artifactual insulin release due to a hormone uptake from the culture medium [65, 66]. Subsequently, several studies focused on discovering the ideal combination of factors for the derivation of definitive endoderm from embryonic stem cells, marking the path for current protocols that represent the paradigm of ongoing pre-clinical studies. In particular D’Amour et al. and Kroon et al. defined *in vitro* stepwise pancreatic-induction protocols that recapitulated each phase of islet cells development from embryonic stem cell to mature endoderm, pancreatic progenitors and, finally, insulin producing beta-cell like cells [67–69]. As a result, C-peptide and insulin secretions were observed in cultures. *In vivo* experiments on immunodeficient diabetic mices revealed that normoglycemia was routinely obtained upon injection of human ESCs-derived beta cells progenitors [70–72]. Notably, the transplantation of ESCs-derived pancreatic progenitors, rather than full differentiated ESCs-derived beta cells, proved to be more effective in restoring normoglycemia by means of the glucose-dependent release of insulin. Progenitor cells might retain a favoured stemness profile for the *in vivo* maturation of functional insulin-producing cells that could provide adequate hyperglycemia responsiveness. The factors that allow the efficient beta-cell differentiation *in vivo* are unknown, but may include key elements that are difficult to reproduce *in vitro,* such as vascularization and interaction with contiguous tissues [73].

However, the transplantation of a mixed population of immature human ESC-(hESC)-derived cells is accompanied by serious safety concerns related to the persistence of the tumorigenic risk. Current studies are being addressed to solve the tumorigenic issues through the sorting of the pancreatic progenitors from the rest of the population [74] and antibody-based methods for the ablation of tumorigenic cells [75].

An interesting alternative to ensure safety and functionality of transplanted beta cell precursors and hESCs-derived precursors, is represented by the use of cell-encapsulation procedures. The enclosure of cells within a barrier would allow diffusion of glucose, nutrients, insulin and block larger molecules, cells and antibodies. The physical selective barrier provided by capsules could avoid the continuous subministration of immunosuppressive drugs, thus ameliorating the quality of life of diabetic patients. Additionally, the capsule would protect the patient from any risk of tumorigenicity and allow the removal of the graft in case of malfunctioning, increasing the recipient safety after transplantation. Breakthrough studies have adopted encapsulation methods to transplant human beta cell progenitors obtained from stem cells. Seung-Hee Lee et al. transplanted human beta cell progenitors into a macroencapsulation device (TheraCyte), durable and biologically inert, which can be transplanted into human patient for a year without adverse effects. The study demonstrated the lasting survival, differentiation and function of human beta cell progenitors in the device, with evidence of cell replication and maturation. This approach eventually resulted in the restoration of blood glucose levels. In addition, the device provided protection from allograft rejection for more than 140 days, suggesting that encapsulated β-cells are invisible to the immune system [76].

## New frontiers: human amniotic epithelial cells

Placenta is the organ responsible for the correct foetal development throughout all the phases of gestation, and is considered a waste product of birth, but its tissues and cells represent an abundant source of stem cells [77, 78]. Four regions of foetal placenta can be distinguished: amniotic epithelial, amniotic mesenchymal, chorionic mesenchymal and chorionic trophoblastic. Cells with variable plasticity are isolated from each region: human amniotic epithelial cells (hAEC), human amniotic mesenchymal stromal cells (hAMSC), human chorionic mesenchymal stromal cells (hCMSC), and human chorionic trophoblastic cells (hCTC). Among all the placental-derived cells, hAECs and hAMSCs are both isolated from the amnion foetal membrane. Various studies showed that hAECs express stem cell markers and retain the ability to differentiate toward all three germ layers, therefore amnion have been proposed as a biological source of stem cells for regenerative medicine [79]. The advantages of using placenta-derived cells can be resumed in the high and worldwide availability of the samples, the non-invasive procedures to collect and handle the tissue/cells, the lack of any ethical issue related to the use of controversial cells, and the low risk of graft-versus-host (GVDH) diseases due to the immune privilege retain by placental tissues [80–83].

Moreover the transplantation of placenta-derived stem cells for cellular therapy fully complies with safety requirements as there is no risk of tumorigenesis (teratomas) [84]. In the last decade several promising studies have been performed in animal and human trials, targeting various diseases [85–90] including diabetes. Wei et al. have shown that human amniotic epithelial cells from the foetal part of the amnion after stimulation with Nicotinamide successfully express insulin mRNA *in vitro* and were capable of normalizing the blood glucose level of streptozotocin-induced diabetic mice for several weeks after cells injection [91]. Recently Okere et al. showed the *in vitro* development of hAECs 3D spheroids: “epi-spheres” were then successfully induced into glucose-responsive insulin producing cells [92]. Another report showed a 7-days *in vitro* differentiation protocol to induce hAECs into definitive endoderm, pancreatic foregut, pancreatic endoderm and, finally, pancreatic endocrine cells [93]. The insulin producing cells derived from hAECs were transplanted in STZ-rodents and after few weeks, a significant and stable amelioration of glycaemia was observed.

## Conclusions

Despite the numerous and encouraging results presented in the last decade, there is still a long road ahead to set a reliable cellular therapy for diabetes. Scientists from all over the world have to set their current and future studies on the basis of two major challenges of cellular therapy: the safety and the efficiency of the transplanted cells. Unfortunately, a cell source providing a robust response after differentiation, is not always considered safe because of transplant-related collateral effects such as immune rejection or, worse, tumorigenesis. Such challenges could be simplified by the adoption of totipotent ESCs in opposition to adult stem cells. ESCs have the greatest stemness potential, hence differentiation potential towards any lineage, any tissue and cell, but their use raises concerns about safety and ethical issues. Although the differentiation protocols have considerably improved their efficiency the transplant of ESCs-derived cells is still associated with the development of malignant tumours due to incomplete differentiation of the clones. Moreover, cells differentiation may lead to secretome changes and phenotype alterations that can elicit the host immune response compelling the patient to an immunosuppressive regimen. In order to bypass the response from the immune system some researchers are developing and testing various devices to encapsulate the transplanted cells within a physical barrier, which could allow the permeability to nutrients, glucose and secreted hormones, and avoid contact with complex molecules, cells and antibodies [76]. Despite hurdles such as material’s biocompatibility, size and porosity, encapsulation systems showed encouraging outcomes with the combination of a mild immunosuppression therapy, which enhanced the functionality of the capsule and increased survival of the transplanted cells within. Furthermore, culture of ESCs requires tools, equipment and resources hardly affordable by most laboratories. The use of the totipotent source is also hampered by ethical issue since their use is forbidden by local jurisdictions in several countries, including most European Nations. In this scenario, a cell therapy based on ESCs-derived cells will unlikely be worldwide available in the near future.

For this reasons an increasing number of scientists are now focusing on different sources of stem cells that possess less stemness potential compared to ESCs but are safer and free of ethical concerns, namely adult stem cells. The pancreas itself is a promising source for stem cells: duct cells, acinar cells and stem cell from islets of Langerhans share the same embryological origin of beta cells; therefore they are good candidates for the de-differentiation and re-programming towards an insulin producing phenotype. The obstacles to the extensive use of this source are represented by the invasive collection procedures, the scarcity of the stem cells, the difficulty in isolating and expanding the culture *in vitro*.

The derivation of insulin producing cells from hepatocytes (trans-differentiation or lateral re-programming), which share with beta cells the same endodermic origin, showed promising outcomes. However, even though these tissues represent an abundant pool of reprogrammable cells, the use of hepatocytes ex vivo is hampered by the shortage of donors, poor *in vitro* expansion capacities, and rapid cell dedifferentiation [46].

Numerous studies described the use of MSC from various tissues, pancreas included, as a widely accessible source of insulin producing cells. The expectations on MSC studies were nurtured by the abundance of cells, the ease in isolation and *in vitro* expansion, and the promising outcomes of various studies showing the differentiation potential towards non-mesodermic lineages. However, *in vivo* assays on animal models did not prove to be as solid as *in vitro* experiments. In most cases transplanted MSC-derived insulin-producing cells failed to afford a stable amelioration in STZ-induced diabetic mice. The controversy concerning *in vivo* potential for MSC cells to differentiate into insulin producing cells is supported by the postulated inability of mesodermic cells to generate functional endocrine pancreatic cells, which could be properly derived only from endodermic progenitors. Further studies are required to improve the *in vitro* pancreatic differentiation of MSC and consequently a stable and responsive production of insulin *in vivo*.

Therefore, scientists are continuously seeking for the optimal characteristics of a stem cell source, which may include: post-transplantation safety, wide availability, non-invasive collection procedure, *in vitro* expandability and differentiation efficiency. Stem cells isolated from the amnion layer of placenta meet most of the mentioned criteria and the adoption of hAECs in cell therapy and regenerative medicine do not raise any ethical concern, unlike the use of ESCs which is restricted or forbidden in most countries [92]. Further *in vitro* evaluation will be necessary to define the therapeutic potential, but there is reason to be optimistic that a sufficient number of efficient insulin producing cells will be available in the near future.

## Abbreviations

AD-MSCs, adipose-derived mesenchymal stem cells; AF-MSCs, amniotic fluid mesenchymal stem cells; BM-MSCs, bone marrow mesenchymal stem cells; hAECs, human amniotic epithelial cells; hAMSCs, human amniotic mesenchymal stromal cells; hCMSCs, human chorionic mesenchymal stromal cells; hCTCs, human chorionic trophoblastic cells; IPF-1/PDX1, insulin promoter facto-1/pancreas and duodenal homebox-1; STZ, streptozotocin; UCB-MSCs, umbilical cord blood mesenchymal stem cells; WJ-MSCs, Wharton’s jelly mesenchymal stem cells
